# Interleukin-2 PET imaging in patients with metastatic melanoma before and during immune checkpoint inhibitor therapy

**DOI:** 10.1007/s00259-021-05407-y

**Published:** 2021-06-02

**Authors:** Pim P. van de Donk, Thijs T. Wind, Jahlisa S. Hooiveld-Noeken, Elly L. van der Veen, Andor W. J. M. Glaudemans, Arjan Diepstra, Mathilde Jalving, Elisabeth G. E. de Vries, Erik F. J. de Vries, Geke A. P. Hospers

**Affiliations:** 1 Department of Medical Oncology, University Medical Center Groningen, University of Groningen, P.O. Box 30.001, 9700 RB Groningen, The Netherlands; 2 Department of Nuclear Medicine and Molecular Imaging, University Medical Center Groningen, University Medical Center Groningen, Groningen, The Netherlands; 3 Department of Pathology and Medical Biology, University Medical Center Groningen, University of Groningen, Groningen, The Netherlands

**Keywords:** Positron emission tomography, T cells, Immunotherapy, Interleukin-2, Melanoma

## Abstract

**Purpose:**

Immune checkpoint inhibitors can induce a T cell–mediated anti-tumor immune response in patients with melanoma. Visualizing T cell activity using positron emission tomography (PET) might allow early insight into treatment efficacy. Activated tumor–infiltrating T cells express the high-affinity interleukin-2 receptor (IL-2R). Therefore, we performed a pilot study, using fluorine-18-labeled IL-2 ([^18^F]FB-IL2 PET), to evaluate whether a treatment-induced immune response can be detected.

**Methods:**

Patients with metastatic melanoma received ~ 200 MBq [^18^F]FB-IL2 intravenously, followed by a PET/CT scan before and during immune checkpoint inhibitor therapy. [^18^F]FB-IL2 uptake was measured as standardized uptake value in healthy tissues (SUV_mean_) and tumor lesions (SUV_max_). Response to therapy was assessed using RECIST v1.1. Archival tumor tissues were used for immunohistochemical analyses of T cell infiltration.

**Results:**

Baseline [^18^F]FB-IL2 PET scans were performed in 13 patients. SUV_mean_ at baseline was highest in the kidneys (14.2, IQR: 11.6–18.0) and liver (10.6, IQR: 8.6–13.4). In lymphoid tissues, uptake was highest in spleen (10.9, IQR: 8.8–12.4) and bone marrow (2.5, IQR: 2.1–3.0). SUV_max_ in tumor lesions (*n* = 41) at baseline was 1.9 (IQR: 1.7–2.3). In 11 patients, serial imaging was performed, three at week 6, seven at week 2, and one at week 4. Median [^18^F]FB-IL2 tumor uptake decreased from 1.8 (IQR: 1.7–2.1) at baseline to 1.7 (IQR: 1.4–2.1) during treatment (*p* = 0.043). Changes in [^18^F]FB-IL2 tumor uptake did not correlate with response. IL-2R expression in four archival tumor tissues was low and did not correlate with baseline [^18^F]FB-IL2 uptake. No [^18^F]FB-IL2-related side effects occurred.

**Conclusion:**

PET imaging of the IL-2R, using [^18^F]FB-IL2, is safe and feasible. In this small patient group, serial [^18^F]FB-IL2-PET imaging did not detect a treatment-related immune response.

**Trial registration:**

Clinicaltrials.gov: NCT02922283; EudraCT: 2014-003387.20

## Introduction

Immune checkpoint inhibitors (ICIs) have demonstrated remarkable efficacy for the treatment of multiple tumor types, including melanoma [1–7]. These monoclonal antibody–based therapies exert their effect by blocking inhibitory ligand-receptor interactions of immune checkpoints, such as cytotoxic T lymphocyte–associated antigen 4 (CTLA-4), programmed cell death protein 1 (PD-1) receptor, or its ligand (PD-L1) [8]. ICI therapy has demonstrated durable and long-lasting responses in a subset of patients. However, a substantial group of patients does not respond to ICI therapy, and it remains challenging to distinguish responders from non-responders early during treatment.

ICIs induce or reinvigorate a T cell–mediated anti-tumor immune response. Also, the presence of tumor-specific T cells in the tumor microenvironment is a key predictive factor for response to ICI therapy [9]. Visualizing these T cells could provide valuable insight into the anti-tumor immune response. For this purpose, we developed N-(4-^18^F-fluorobenzoyl)-interleukin-2 ([^18^F]FB-IL2), a clinical-grade fluorine-18-labeled IL-2 PET tracer [10].

IL-2 is a 15-kDa cytokine that plays an important role in the cellular immune response. Its primary function involves stimulation of growth, proliferation, activation, and differentiation of T cells [11]. IL-2 induces its effects by binding to transmembrane IL-2 receptors (IL-2Rs). The high-affinity IL-2R, consisting of three subunits (CD25, CD122, and CD132), is primarily present on activated effector T cells and regulatory T cells (T_reg_), whereas the low-affinity IL-2R, consisting of two subunits (CD122 and CD132), is generally found on naïve T cells and natural killer cells [12].

Quite recently, single-photon emission computed tomography (SPECT) imaging of the IL-2R has been performed in five patients with metastatic melanoma [13]. However, PET imaging provides better spatial resolution and allows for more accurate quantification of tracer uptake in tumor lesions and other tissues. Therefore, we developed [^18^F]FB-IL2 as a PET tracer to visualize the migration of activated T cells [14, 15].

Based on the promising results obtained in our preclinical studies, we performed a clinical feasibility study in patients with metastatic melanoma to determine the biodistribution and tracer kinetics of [^18^F]FB-IL2, and to evaluate whether serial [^18^F]FB-IL2 PET imaging can detect an ICI-induced immune response in tumor lesions.

## Materials and methods

### Study population

For this study, patients with ICI treatment-naïve metastatic melanoma (stage IV) were included. Patients were eligible if they had measurable disease according to RECIST v1.1, age ≥ 18 years, Eastern Cooperative Oncology Group (ECOG) performance status of 0–1, and adequate hematologic and end-organ function. The main exclusion criteria were pre-existing auto-immune disease, treatment with immunosuppressive medication, and symptomatic or unstable brain metastases. All patients provided written informed consent.

### Study design

This single-center, open-label, non-randomized imaging study was performed at the University Medical Center Groningen (UMCG), the Netherlands. The study was approved by the Medical Ethical Committee of the UMCG, delegated by the Central Committee on Research Involving Human Subjects, and was registered at ClinicalTrials.gov (identifier NCT02922283).

All patients were treated with an ICI according to the standard treatment schedules for a maximum of 2 years. Patients received either pembrolizumab every 3 weeks, nivolumab every 2 weeks, or the combination of ipilimumab and nivolumab. This combination consisted of 4 cycles of ipilimumab and nivolumab every 3 weeks, followed by nivolumab monotherapy every 2 weeks [16].

### [^18^F]FB-IL2 PET scans

Clinical-grade [^18^F]FB-IL2 was prepared according to Good Manufacturing Practice (GMP) standards, as previously described [10]. Before and during ICI therapy, patients received an intravenous bolus injection of approximately 200 MBq (range 116–213 MBq) [^18^F]FB-IL2 in 5 min. The injected mass dose of [^18^F]FB-IL2 was always less than 50 μg. Due to the limit of quantitation of the quality control procedure, injected doses < 37 μg could not be accurately measured. PET image acquisition followed 60 min after tracer injection on a Biograph 64 slice mCT camera (Siemens Medical Systems, Knoxville, TN, USA). Total body PET scans (head to toe; 12-bed positions, 3 min per bed position), accompanied by a low-dose CT scan, were acquired. Six venous blood samples (sodium heparin) of 7 mL each were collected at 5, 10, 15, 25, 40, and 60 min post-injection for metabolite analysis and assessment of tracer kinetics in plasma. Radioactivity was measured in 250 μL plasma with a calibrated well-type gamma counter (LKB Instruments). Radioactivity was expressed as standardized uptake value. The degradation of [^18^F]FB-IL2 into small radioactive protein fragments was determined using a trichloroacetic acid precipitation assay [15]. For the on-treatment PET series, patients were initially scanned after 6 weeks of ICI therapy. In the first three patients, no increase in tracer uptake was observed in responding tumor lesions. Therefore, the protocol was amended to change the imaging timepoint of the on-treatment PET scan to 2 weeks after initiation of ICI therapy.

### [^18^F]FB-IL2 PET data analysis

All PET scans were reconstructed according to EARL and analyzed using Syngo.via VB20 software (Siemens) [17]. For quantification of [^18^F]FB-IL2 tumor uptake, volumes of interest (VOI) were manually drawn, using the PET images for lesions visually discernible from the background and the low-dose CT images for PET-negative lesions. PET-negative lesions, surrounded by high physiological uptake, were excluded from further analysis. Furthermore, uptake was determined in healthy tissues (brain, lung, left ventricle, thoracic aorta, thigh muscle, kidney cortex, bone cortex, liver, thyroid, and parotid glands) as well as in lymphoid tissues (axillary and inguinal lymph nodes, spleen, bone marrow, and tonsils). Tracer uptake was corrected for body weight and injected dose and is expressed as standardized uptake values (SUV_max_ for tumor lesions and SUV_mean_ for normal tissues) in line with EANM guidelines for ^18^F tracers in tumors [18].

### Other study assessments

Formalin-fixed and paraffin-embedded archival tumor samples, obtained within 5 weeks before the first PET scan, were used for immunohistochemical (IHC) analysis of T cells in the tumor microenvironment. CD3 (2GV6, Ventana), CD4 (SP35, Ventana), and CD8 (C8/144B, DAKO) were stained using an automated IHC platform (Roche Ventana BenchMark Ultra) and standard diagnostic procedures. For CD25 (IL-2R alpha chain) staining, microwave antigen retrieval was performed in 0.1 M Tris/HCl at pH 9.0, and monoclonal CD25 antibody (NCL-CD25-305, Novocastra) was used at 1:50 dilution. Secondary and tertiary peroxidase-conjugated antibodies were visualized by diaminobenzidine staining reaction. Slides were scanned on a Philips IntelliSite scanner, and representative images of 0.13 mm^2^ each were scored. For each sample and each stain, 3 images were obtained from the central tumor area and 3 images from the invasive front. The stains were performed on consecutive slides and scoring of the number of positive cells was done on the same areas for all 4 markers.

Response to therapy was evaluated according to the RECIST v1.1 and iRECIST criteria [19, 20]. Contrast-enhanced chest-abdominal CT scans were performed at weeks 12 and 16, and after that as part of routine patient care.

Adverse events were assessed at each outpatient visit, using the National Cancer Institute Common Terminology Criteria for Adverse Events version 4.0 [21], and reported until the end of the study (week 16). Vital signs (blood pressure and heart rate) were measured before, 10 min after the injection of [^18^F]FB-IL2, and immediately after the PET-CT scan. Patients remained under observation for 120 min after tracer injection.

### Statistical analyses

Patients were evaluable for biodistribution analysis if they underwent at least one [^18^F]FB-IL2 PET scan. An assessment of the normality of data was performed using the Shapiro-Wilk test. Differences in tracer uptake between the baseline and on-treatment [^18^F]FB-IL2 PET scans were analyzed using a Wilcoxon signed-rank test. Correlations between parameters were calculated using the Spearman correlation test. *P* values < 0.05 were considered significant. PET data is expressed as median with interquartile range (IQR). Analyses were performed using IBM SPSS Statistics software version 22 for Windows.

## Results

Nineteen patients were enrolled, of whom six were not evaluable as a result of tracer production failure before the first tracer administration. These PET scans were not rescheduled, as delay of treatment was deemed inappropriate for these patients. These patients were excluded from further trial participation. Two patients could not undergo a second IL-2 PET scan due to tracer production failure before the second tracer administration. For one patient, the second tracer injection was postponed to 4 weeks after treatment initiation. In total, 13 patients were scanned at baseline and 11 patients underwent both [^18^F]FB-IL2 PET scans (Table 1). None of the patients experienced infusion reactions or other [^18^F]FB-IL2-related adverse effects.

**Table 1:** Patient characteristics

Number of patients	13
Median age (years)	67
Sex, *n* (%):	
- Male:	9 (69)
- Female:	4 (31)
ECOG performance status, *n* (%):	
- 0	10 (77)
- 1	3 (23)
Number of [^18^F]FB-IL2-PET scans per patient, *n* (%):	
- 1	2 (15)
- 2	11 (85)
Immunotherapy regimen, *n* (%):	
- Nivolumab	5 (38)
- Pembrolizumab	7 (54)
- Ipilimumab + nivolumab	1 (8)
RECIST v1.1 response, *n* (%):	
- Complete response	1 (10)
- Partial response	1 (10)
- Stable disease	2 (20)
- Progressive disease	6 (60)

Abbreviations used: *ECOG*, Eastern Cooperative Oncology Group; *RECIST*, Response Evaluation Criteria in Solid Tumors

### Biodistribution

**Fig. 1:**
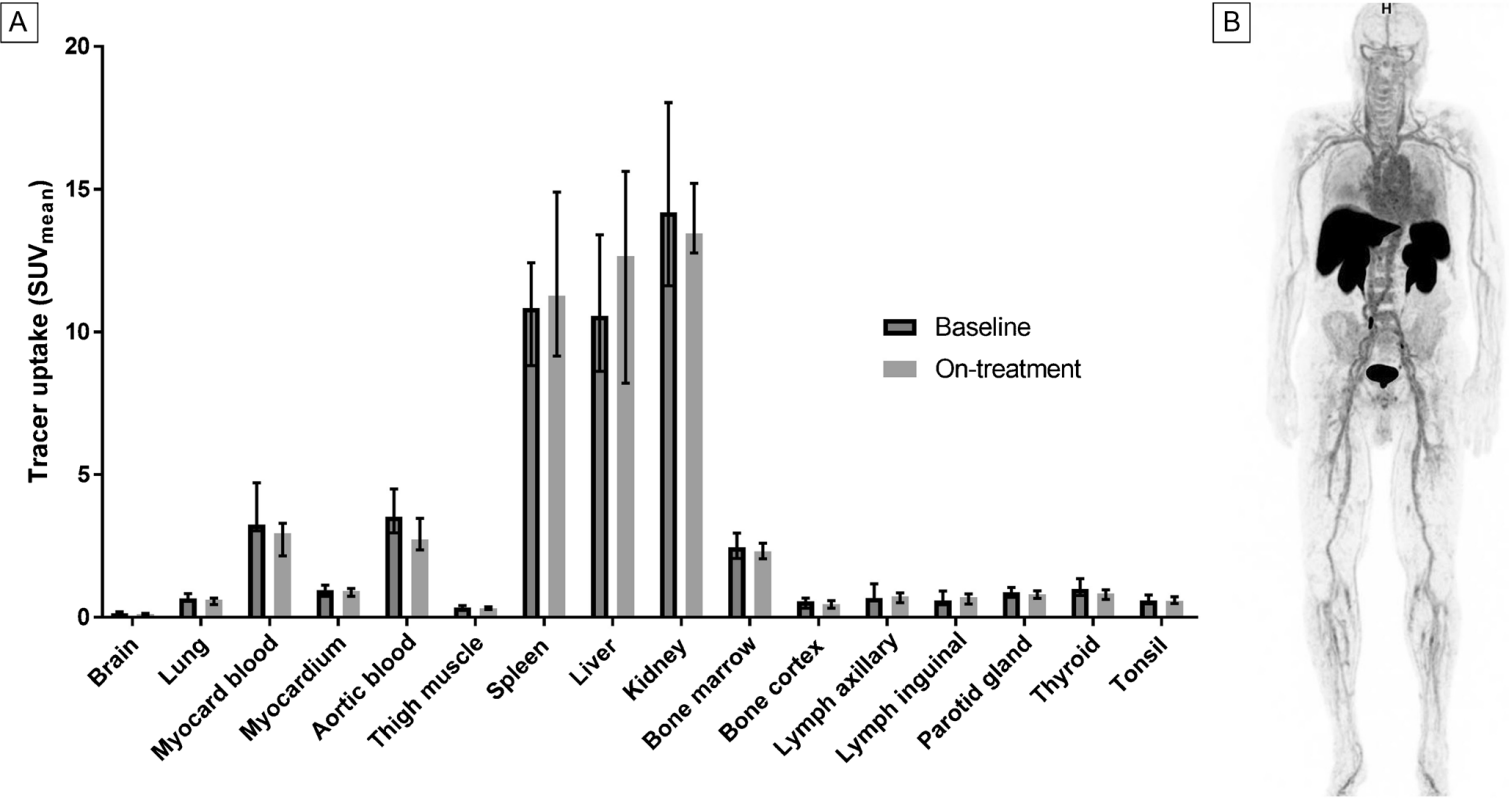
Biodistribution of [^18^F]FB-IL2. **A** Median tracer uptake of healthy tissues, expressed as SUV_mean_ with interquartile range on the baseline [^18^F]FB-IL2-PET scans (*n* = 13) and on-treatment [^18^F]FB-IL2-PET scans (*n* = 11). **B** Example of a maximum intensity projection of the [^18^F]FB-IL2-PET scan

**Fig. 2:**
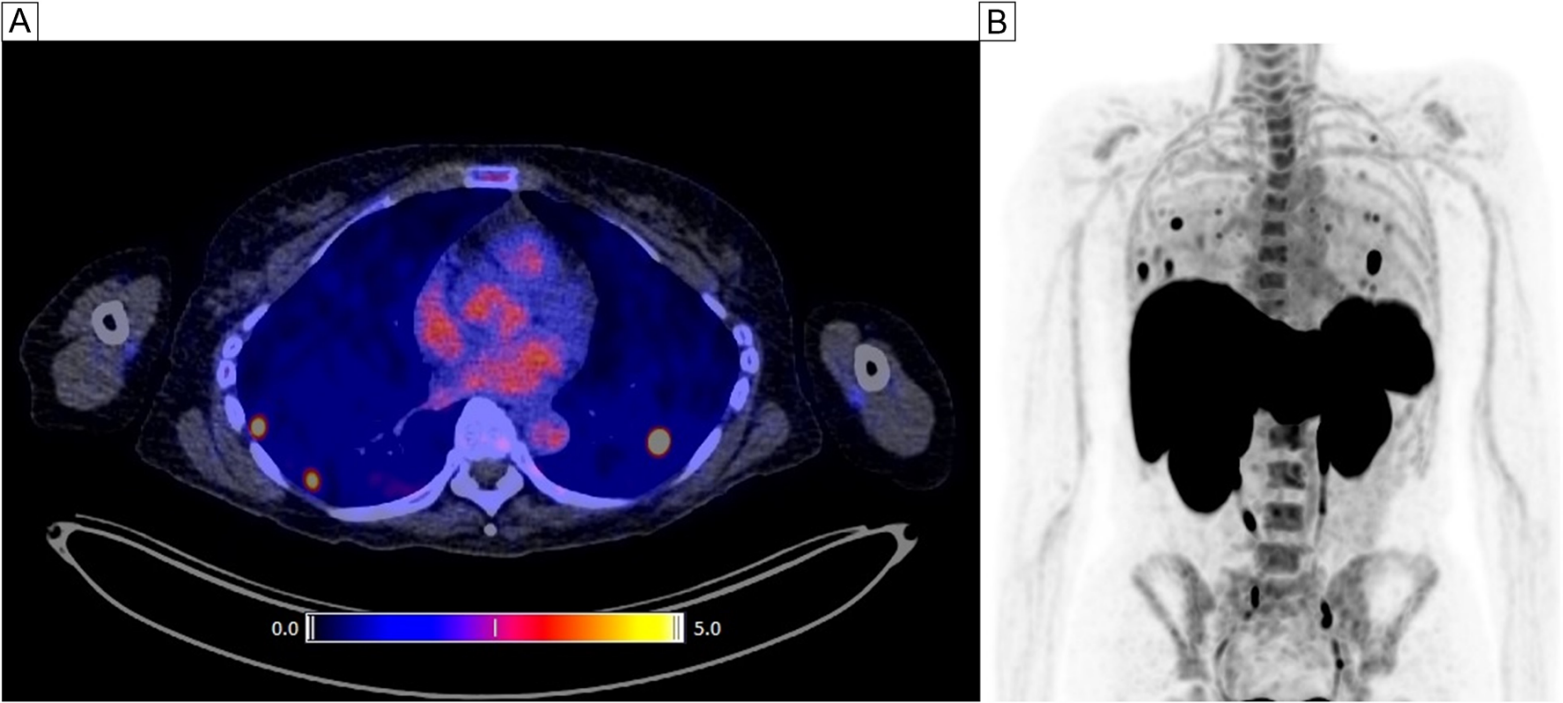
Example of high tracer uptake in the lungs. **A** Transversal PET/CT image of three regions showing high [^18^F]FB-IL2 uptake. **B** Maximum intensity projection of the same patient showing multiple areas of high tracer accumulation in the lungs

The biodistribution of [^18^F]FB-IL2 was evaluated in healthy tissues at baseline (*n* = 13 patients) and on-treatment (*n* = 11 patients). The highest tracer uptake at baseline was observed in the kidneys (SUV_mean_ 14.2, IQR: 11.6–18.0) and liver (SUV_mean_ 10.6, IQR: 8.6–13.4). In lymphoid tissues, there was clear uptake in the spleen (SUV_mean_ 10.9, IQR: 8.8–12.4) and bone marrow (SUV_mean_ 2.5, IQR: 2.1–3.0). Lymph nodes with a normal diameter showed low tracer uptake. Other normal tissues also showed low tracer uptake (Fig. 1). During ICI treatment, the biodistribution of [^18^F]FB-IL2 was comparable to the baseline values, with the highest tracer uptake in the kidneys (SUV_mean_ 13.5, IQR: 2.4), liver (SUV_mean_ 12.7, IQR: 7.4), and spleen (SUV_mean_ 11.3, IQR: 5.7) (Fig. 1). However, a decrease in tracer uptake was observed in the blood pool (myocardial: − 11%, *P* = 0.021; aortic: − 29%, *P* = 0.016), lung tissue (− 8%, *P* = 0.021), and thyroid (− 21%, *P* = 0.028). In three patients, high focal tracer uptake was seen in lung tissue (Fig. 2). However, no anatomical abnormality for this uptake was found in these regions on the low-dose CT scan or subsequent diagnostic CT scans, and patients did not experience any respiratory symptoms.

### Imaging of tumor lesions

**Fig. 3:**
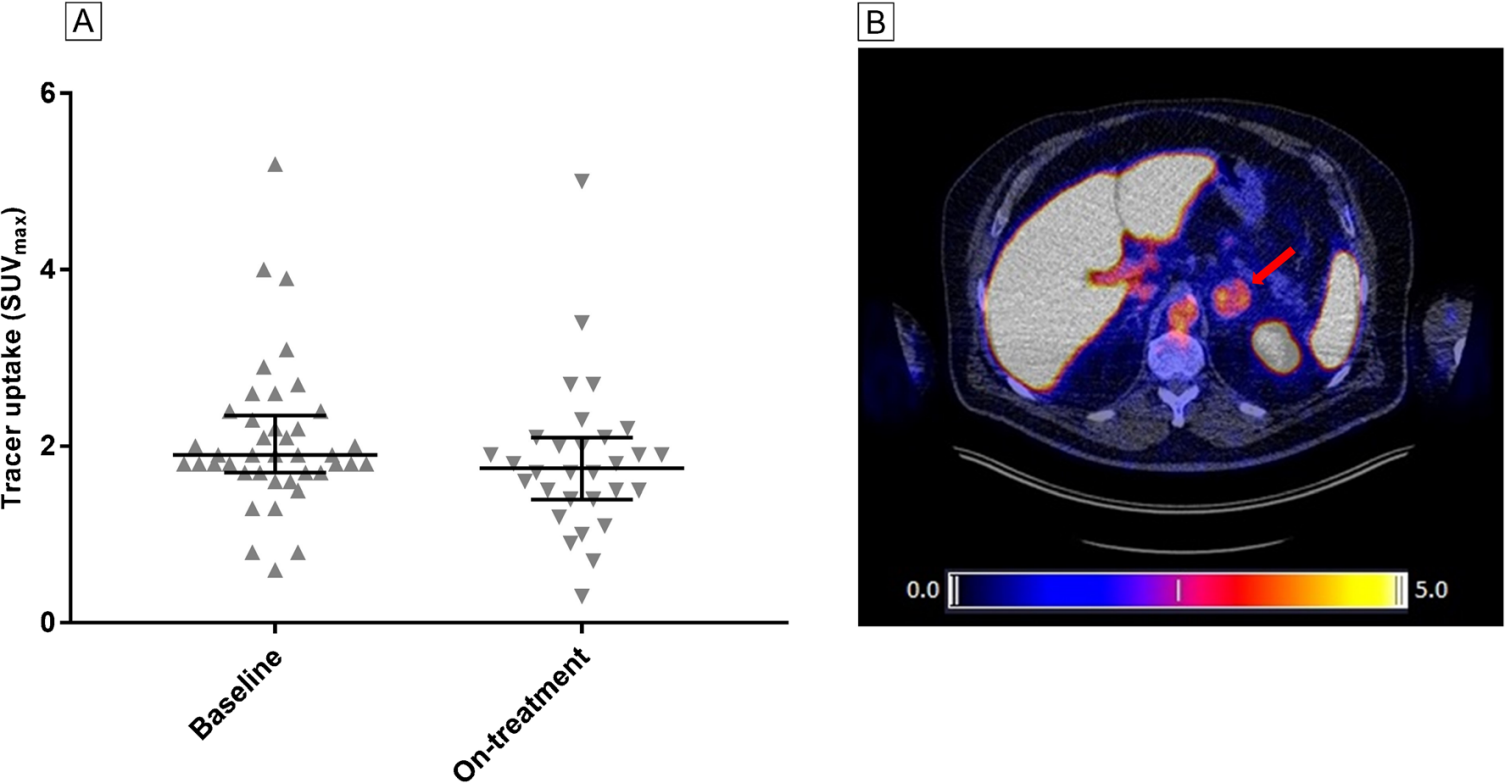
Uptake of [^18^F]FB-IL2 in tumor lesions. **A** Tracer uptake (SUV_max_) in individual tumor lesions per imaging timepoint, horizontal bars represent median with interquartile range. **B** Example of transversal PET/CT image of an adrenal gland metastasis showing [^18^F]FB-IL2 uptake (SUV_max_ of 5.2)

[^18^F]FB-IL2 PET imaging was able to visualize tumor lesions in melanoma patients, although tumor uptake of [^18^F]FB-IL2 was generally low (Fig. 3). Median tracer uptake in tumor lesions at baseline (13 patients, 41 tumor lesions) was SUV_max_: 1.9 (IQR: 1.7–2.3). Serial imaging in 11 patients with 30 tumor lesions showed a decrease in [^18^F]FB-IL2 tumor uptake from SUV_max_ 1.8 (IQR: 1.7–2.1) at baseline to SUV_max_ 1.7 (IQR: 1.4–2.1) during treatment (*P* = 0.043).

### Plasma kinetics

**Fig. 4:**
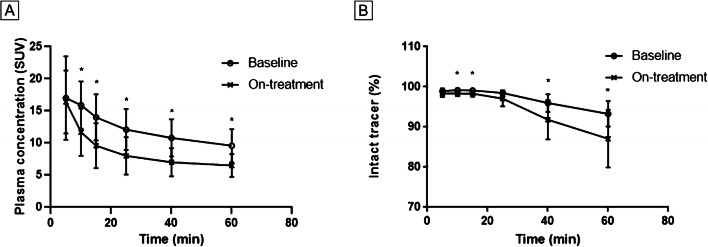
Analyses of [^18^F]FB-IL2 in plasma. **A** Plasma activity over time, expressed as standardized uptake value (mean ± standard deviation). **B** Tracer integrity over time, expressed as the percentage of intact tracer (mean ± standard deviation).*P < 0.05

Tracer kinetics in venous plasma was evaluated in 5 patients (Fig. 4), showing an exponential decrease in radioactivity in plasma over time. The clearance of radioactivity from plasma was faster during the follow-up scan than at baseline, resulting in a significantly lower plasma radioactivity concentration from 10 min onward (~ 30%, *P* < 0.05). The percentage intact tracer in plasma was also significantly lower at follow-up than at baseline (e.g., − 7% at 60 min, *P* = 0.07).

### Immunohistochemistry

**Fig. 5:**
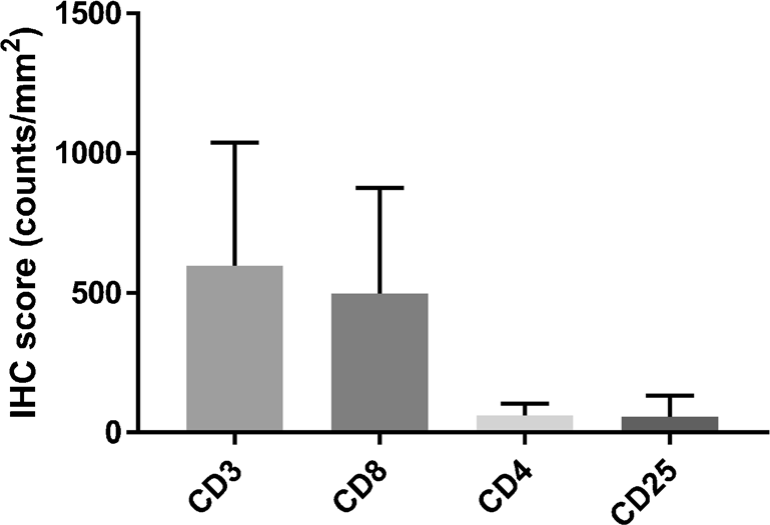
Immunohistochemistry for T cell markers on archival tumor tissue samples. IHC scores are expressed as mean counts/mm^2^ with standard deviation. Abbreviation used: IHC, immunohistochemistry

Trial participation provided no additional benefits for the patients; therefore, it proved difficult to obtain fresh tumor biopsy samples. However, archival tumor tissues of four patients were available for analysis of T cell subsets (Fig. 5). CD25 expression on immune cells (mean: 55, range: 1–169 counts/mm^2^) was low compared to CD3 (mean: 598, range: 46–957 counts/mm^2^) and CD8 (mean: 498, range: 37–820 counts/mm^2^) expression. CD25 staining will visualize both cell surface and intracellular CD25. However, CD25 staining was largely restricted to the cell surface. No correlation was seen between these IHC markers and mean tracer uptake in tumor lesions at baseline.

### Response to therapy

For response evaluation, 19 target lesions were defined for ten evaluable patients according to the RECIST v1.1 criteria. Best overall responses were one complete response, one partial response, and two patients experienced stable disease (Table 1). Three patients did not meet the criteria for response assessment according to RECIST. Mean tracer uptake of tumor lesions at baseline did not correlate with RECIST response to therapy. Also, the change from baseline in tumor tracer uptake (SUV_max_) did not correlate with RECIST response to therapy.

## Discussion

In this first-in-human study, we demonstrate that [^18^F]FB-IL2 PET imaging is a safe and non-invasive imaging modality for whole-body visualization of the IL-2R.

This study adds to previous work using IL-2-based molecular imaging probes. SPECT imaging of the IL-2R, using ^99m^Tc-labeled interleukin-2 (^99m^Tc-HYNIC-IL2), has been performed previously in patients receiving ICI therapy [13]. In a small study, five patients with melanoma were imaged at baseline and three of these patients completed repeat imaging after 12 weeks of ICI therapy. The study demonstrated heterogeneous tracer accumulation in tumor [13]. However, no data is presented on tracer biodistribution or uptake in lymphoid tissues. Here we present data of 13 patients, of which 11 completed repeat [^18^F]FB-IL2 PET imaging. [^18^F]FB-IL2 uptake was quantified in tumor lesions, as well as in normal tissues to assess the biodistribution of [^18^F]FB-IL2 at baseline and during treatment with an ICI.

High [^18^F]FB-IL2 uptake was observed in lymphoid tissues, such as the spleen and bone marrow. This is highly suggestive of IL-2R-specific tracer accumulation in the lymphoid system. High [^18^F]FB-IL2 uptake was also observed in the major excretion organs kidneys and liver [22, 23]. The overall tracer biodistribution at baseline was very similar compared to the on-treatment scan, with a minor decrease in tracer uptake in the blood pool, lung tissue, and thyroid observed for the latter. In three patients, we observed high focal uptake in the lungs. These patients had no pulmonary complaints or anatomic abnormalities justifying this uptake on conventional imaging modalities. A possible explanation for the focal uptake in the lungs could be the formation of micro-aggregates of [^18^F]FB-IL2 alone, or together with human serum albumin from the tracer solution. These micro-aggregates could have been trapped in the peripheral microvasculature of the lungs, resulting in the observed focal uptake. IL-2 tends to aggregate after reconstitution in water, which was one of the issues that had to be overcome during tracer development [10]. The formation of aggregates was not observed during the quality control of the tracer, but it cannot be excluded that it occurred afterwards.

Tracer uptake in tumor lesions at baseline was relatively low (median SUV_max_: 1.9), which is consistent with the low CD25 expression detected in the archival tumor tissues. However, the, albeit modest, decrease in tumor tracer uptake during treatment was unexpected. We hypothesized that ICI therapy would induce an immune response in some of the patients, which would result in higher T cell numbers and higher IL-2R expression due to T cell activation. Multiple studies have reported an increase in tumor–infiltrating T cells during the first few weeks of treatment in tumor tissue samples [9, 24–29]. After three patients, the on-treatment imaging timepoint was changed from 6 to 2 weeks, since the transient upregulation of the IL-2R on T cells following ICI therapy could take place earlier and might have been missed. However, imaging after 2 weeks of treatment yielded similar results. Some studies have found CD25 expression in tumors to be restricted mainly to T_regs_ and demonstrate no significant change over time in tumor tissues following ICI therapy [24, 25, 30]. This could mean that [^18^F]FB-IL2 accurately depicts CD25 expression in the tumor microenvironment.

Another possible explanation for the decrease in tracer uptake in tumor lesions could be antidrug antibody (ADA) formation. This does occur for many biological drugs and can lead to altered pharmacokinetics or inactivation of these drugs [31]. Aldesleukin is the main component of this PET tracer, only modified by the [^18^F]fluorobenzoyl group. During treatment with aldesleukin, 70.8% of patients produce ADAs [32]. Whether ADA formation influenced the pharmacokinetics of [^18^F]FB-IL2 remains unknown, as ADA testing was not part of this study. However, we did observe higher metabolite formation and a significant decrease in plasma activity after the second [^18^F]FB-IL2 injection (Fig. 4). This could also explain the decrease in blood pool activity and tracer uptake in some of the normal tissues observed in the on-treatment PET series. A final explanation for the slight decrease in tumor tracer uptake during ICI treatment might becompetition with endogenous IL-2 for binding to the IL-2R. ICI therapy can activate T cells, and this may subsequently result in higher IL-2 production by CD4+ T-helper cells in the tumor microenvironment. This would reduce the number of available binding sites for [^18^F]FB-IL2. Future studies should include more extensive blood and tissue collection to address the abovementioned hypotheses.

Imaging of the IL-2R, using [^18^F]FB-IL2, will only visualize a fraction of the immune cells present in the tumor microenvironment. Additionally, the high-affinity IL-2R CD25 is present on T_regs_ as well as activated CD8+ T cells. Consequently, [^18^F]FB-IL2 PET imaging will visualize both immunosuppressive and immunostimulatory cells in the tumor microenvironment. Currently, multiple PET tracers are being investigated within the field of immuno-oncology. These include tracers targeting immune checkpoints, such as PD-L1 and PD-1 [33, 34], and tracers targeting specific immune cell subsets, such as CD8+ T cells [35, 36]. Each of these tracers might provide more insight into a specific aspect of the tumor microenvironment. Combining the information of multiple scans could reveal a more complete picture of the tumor microenvironment and could possibly be used to differentiate responders from non-responders [37, 38].

Currently, effort is being put into optimizing the imaging performance of IL-2-based PET tracers and simplifying their production to obtain more reliable yields. Several new and improved IL-2-based PET tracers have already been produced and show promising results in preclinical studies [39].

In summary, the results of this study show that PET imaging of the IL-2R, using [^18^F]FB-IL2, is safe and feasible in patients with metastatic melanoma during ICI therapy. However, in this small patient group, serial [^18^F]FB-IL2-PET imaging was unable to detect a treatment-related immune response. Whether this observation is the result of insufficient infiltration of activated T cells, or insufficient sensitivity of the imaging technique to detect the treatment-induced immune response, cannot be concluded from our study and therefore still remains to be investigated.

## Data Availability

The datasets generated during and/or analyzed during the current study are available from the corresponding author on reasonable request.
